# HLA-DMB restricts human T-cell leukemia virus type-1 (HTLV-1) protein expression via regulation of ATG7 acetylation

**DOI:** 10.1038/s41598-017-14882-z

**Published:** 2017-10-31

**Authors:** Jie Wang, Di Song, Yanzi Liu, Guangjian Lu, Shuai Yang, Lu Liu, Zhitao Gao, Lingling Ma, Zhixiang Guo, Chenguang Zhang, Hui Wang, Bo Yang

**Affiliations:** 1 Henan Collaborative Innovation Center of Molecular Diagnosis and Laboratory Medicine, School of Laboratory Medicine, Xinxiang Medical University, Xinxiang, 453003 Henan Province China; 2 Henan Key Laboratory of immunology and targeted drugs, Xinxiang Medical University, Xinxiang, 453003 Henan Province China; 3 Department of Laboratory Medicine, the Third Affiliated Hospital of Xinxiang Medical University, Xinxiang, 453003 Henan Province China; 4 Clinical Laboratory, the First Affiliated Hospital of Xinxiang Medical University, Weihui, 453100 Henan Province, China; 5 Department of Immunology/Department of Bio-therapeutic, Institute of Basic Medicine, School of Life Sciences, PLA Medical School, Chinese PLA General Hospital, 28 Fuxing Road, Beijing, 100853 China; 6 Xinxiang assegai medical laboratory institute, Xinxiang, 453003 China

## Abstract

The roles of autophagy in viral infection are complicated. While autophagy has been shown to function in host antiviral defense by eliminating intracellular viruses and regulating adaptive immunity, several viruses have evolved molecular mechanisms to get benefits from it. The deltaretrovirus human T-cell leukemia virus type-1 (HTLV-1) has been reported to profit its replication from enhancing autophagosome accumulation. Here, we reported that HLA-DMB (generally referred to here as DMB), the beta chain of the non-classical MHC-II protein HLA-DM, had strong expression in HTLV-1-transformed T-cell lines and could be induced in Hela, PMA-differentiated THP1 (PMA-THP1) or primary human monocytes by HTLV-1 infection. Immunoblot and real-time PCR assays demonstrated that overexpression of DMB decreased HTLV-1 protein expression while the knockdown of DMB increased HTLV-1 protein expression. Immunoblot and confocal microscopy assays indicated that overexpression of DMB decreased HTLV-1 induced autophagosome accumulation while the knockdown of DMB yielded the opposite effects. Coimmunoprecipitation and immunoprecipitation experiments suggested DMB interacted with autophagy-related gene (ATG) 7 and increased the acetylation of ATG7. Taken together, these results suggested DMB modulated HTLV-1 protein expression through regulation of autophagosome accumulation and our findings suggested a new mechanism by which the host cells defended against HTLV-1 infection.

## Introduction

Human T-cell leukemia virus type-1 (HTLV-1), the first retrovirus discovered to be linked with human diseases^1,2^, infects approximately 10~20 million people worldwide^3^. While most infected individuals are asymptomatic carriers (ACs) of the virus, 3~5% of infected individuals develop a malignancy of CD4+ T cells known as Adult T cell leukemia (ATL) several decades after infection and less than 50% of the ATL patients survive more than one year^4,5^. HTLV-1 also causes a severe neurological disorder designated HTLV-1-associated myelopathy/tropical spastic paraparesis (HAM/TSP) and other inflammatory diseases such as HTLV-1 uveitis^6^.

Autophagy, characterized by the formation of double-membrane vesicles called autophagosomes and subsequent lysosome-based degradation of damaged or excess cellular components, plays an important role in maintaining homeostasis^7,8^. Autophagy is initiated at the isolation membrane, usually from endoplasmic reticulum (ER) membranes, and autophagosome formation is dependent on the so-called autophagy-related gene (ATG) products^9^. Till now, 40 ATG proteins have been identified in yeast and many mammalian homologs for these have been found^10^. However, only half of these are essential for formation of canonical autophagosomes, including ATG1-10, 12–14, 16–18, 29, and 31^11^. Central to canonical autophagy are two ubiquitination-like conjugation systems, ATG12 conjugation system and the microtubule-associated protein 1-light chain 3 (LC3)/ATG8 lipidation system. Both ATG12 and ATG8 are activated by the same E1-like enzymes called ATG7^12^. In the ATG12 conjugation system, ATG7 facilitates the conjugation of ATG12 to ATG5, forming the ATG12-ATG5 conjugate^13^. In the LC3 lipidation system, activated LC3-I is transferred to ATG3 and finally conjugated to phosphatidylethanolamine (PE)^14^. This LC3-PE conjugate is known as LC3-II and is one of the most accepted markers of autophagy now^15^.

Autophagy can be stimulated by nutrient deprivation, growth factor withdrawal, and other signals, including ER stress, oxidative stress, and immune cell activation^16^. Importantly, it is becoming increasingly clear that autophagy is activated upon viral infection^17^. Depending on the virus and the host cell, autophagy can have different effects during viral infection, either as an innate host antiviral defense mechanism or as a pro-viral process^18^. As an integral part of immune system, autophagy has been shown to function in host antiviral defense by limiting viral replication, influencing viral antigens presentation or targeting virions and virus components for autophagic degradation^19–22^. Conversely, certain viruses have evolved diverse mechanisms to exploit the autophagy system for their replication^23–26^. For example, autophagy proteins (i.e., Beclin-1, ATG4B, ATG5, and ATG12) are proviral factors required for translation of incoming hepatitis C virus (HCV) RNA and, therefore, for establishment of productive infection^27^. In addition, recent work has demonstrated that HTLV-1 infection increases the accumulation of autophagosomes and that this accumulation benefits viral replication^28^, although detailed mechanisms remain to be clarified.

Here we demonstrated that HLA-DMB (generally referred to here as DMB), the beta chain of the non-classical MHC-II protein HLA-DM, was induced by HTLV-1 infection and suppressed HTLV-1 protein expression. We showed that DMΒ inhibited the accumulation of autophagosomes during HTLV-1 infection, which was important for HTLV-1 replication. Further study indicated that DMB was associated with ATG7, one of the core autophagy proteins essential for canonical autophagy, and increased its acetylation. Collectively, our findings may shed some new lights on autophagy regulation and contribute to our understanding of the host defenses against HTLV-1 infection.

## Results

### DMB expression is induced by HTLV-1 infection

**Figure 1:**
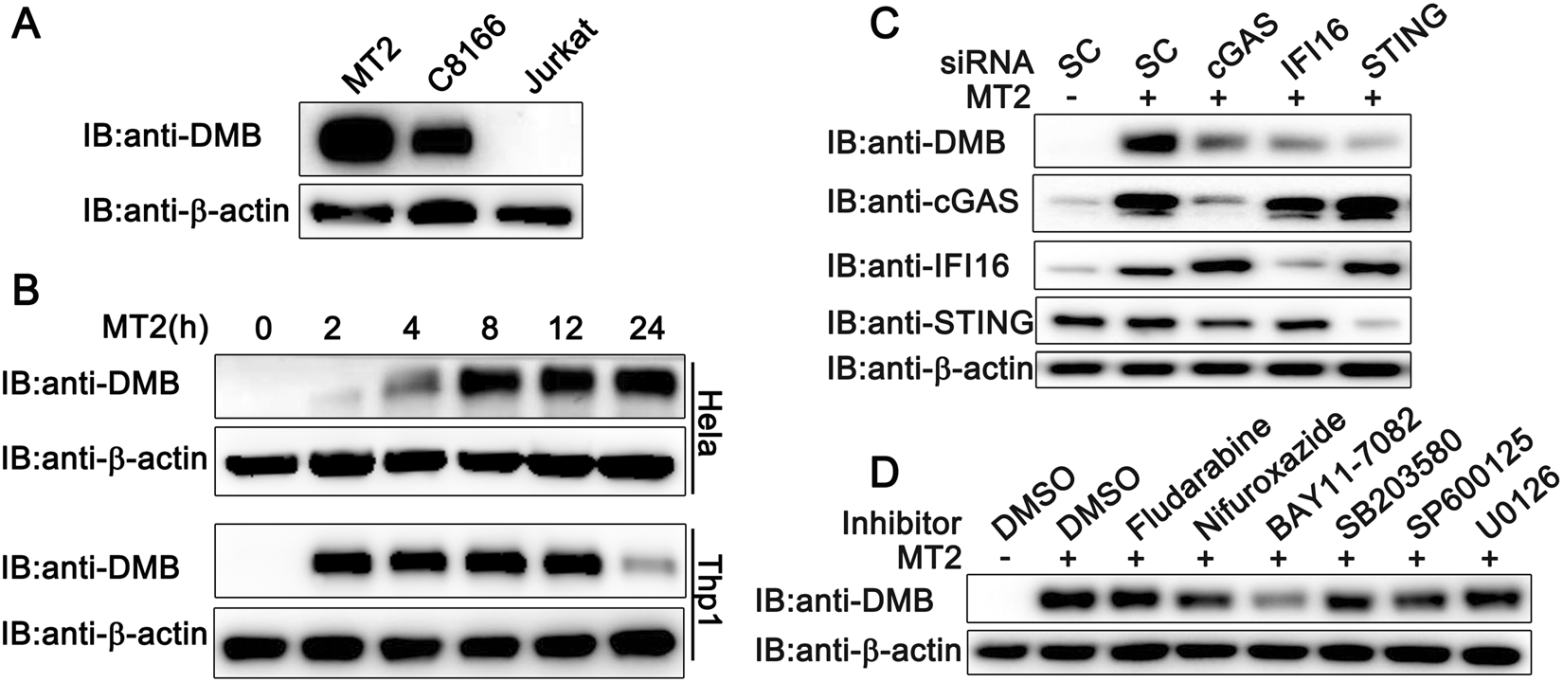
DMB expression associated with HTLV-1 infection. (**A**) MT2, C8166 and Jurkat cells were lysed and analyzed with an anti-DMΒ antibody. (**B**) Hela cells (top) or PMA-THP1 cells (bottom) were co-cultured with MT2 cells for indicated time. Afterwards, the cells were washed with PBS three times to remove MT2 cells and lysed for immunoblot assays. (**C**) Hela cells were transfected with SC or indicated siRNA. At 24 h after transfection, the cells were co-cultured with MT2 cells for another 24 h. Then the cells were washed with PBS three times to remove MT2 cells and lysed for immunoblot analyses. (**D**) Hela cells were treated with DMSO, Fludarabine (10 uM), Nifuroxazide (25 ug/mL), BAY11-7082 (10uM), SB203580 (5 uM), SP600125 (10uM) or U0126 (1 uM) and co-cultured with MT2 cells for 18 h. Then the cells were washed with PBS three times to remove MT2 cells and lysed for immunoblot analyses. β-actin was used as a loading control in the immunoblot assays. The data were representative of three independent experiments.

DMΒ colocalizes and engages in MHC-class-II-antigenic-peptide complexes^29^, which are expressed constitutively in B cells and could be induced by some stimuli in other cell types such as monocytes and T cells^30^. All the above cell types could be infected by HTLV-1^31^. Thus, it seemed reasonable for us to explore the relationship between DMΒ and HTLV-1 infection. We first examined DMΒ expression in HTLV-1 infected cells. We compared the expression of DMΒ in non-infected Jurkat T-cell line as well as in HTLV-1-transformed T-cell lines (C8166 and MT2). Immunoblot assays indicated that the HTLV-1-positive MT2 and C8166 cells showed strong expression of endogenous DMΒ protein, whereas the HTLV-1-negative Jurkat cells did not (Fig. 1A). We next examined DMΒ expression in HTLV-1 infected Hela and PMA-differentiated THP1 (a human macrophage-like cell line, PMA-THP1) cells. Hela and PMA-THP1 cells were co-cultured with MT2 cells and immunoblot assays demonstrated that DMΒ expression was induced in MT2-co-cultured cells (Fig. 1B).

We tried to investigate the molecular mechanisms by which DMB expression was induced in HTLV-1 infected cells. Because the reverse transcription intermediates (RTIs) of retroviruses could be recognized by DNA sensors, we speculated that DMB might be induced by cytosolic DNA sensor pathways. It has been clarified that HTLV-1 RTIs interact with STING and HTLV-1 infection triggers a robust anti-viral innate immune response in primary monocytes^32^. Although the exact roles of cGAS and IFI16 in HTLV-1 infection were unclear, it is known that IFI16 and cGAS could interact with RTIs generated by some other retroviruses and recruit STING for downstream signal transduction^33,34^. So, we first investigated the roles of cGAS, IFI16 and STING in DMB induction during HTLV-1 infection. Interestingly, after cGAS, IFI16 or STING knockdown, the DMB expression was decreased markedly in Hela cells after HTLV-1 co-culture (Fig. 1C), suggesting that the DMB induction by HTLV-1 infection dependent on cytosolic DNA sensors. Then we tried to figure out the downstream signaling pathways involved in this process. We treated Hela cells with Fludarabine (for STAT1 inhibition), Nifuroxazide (for STAT1/3/5 inhibition), BAY11–7082 (for NF-κB inhibition), SB203580 (for p38 MAPK inhibition), SP600125 (for JNK1/2/3 inhibition) or U0126 (for MEK1/2 inhibition) and the DMB expression was examined after MT2 infection. The results suggested NF-κB was involved in DMB induction by HTLV-1 infection (Fig. 1D). Taken together, our data suggested that the expression of DMΒ could be induced by HTLV-1 infection and NF-κB was critical to HTLV-1 triggered DMB production.

### DMB expression decreases HTLV-1 protein expression

**Figure 2:**
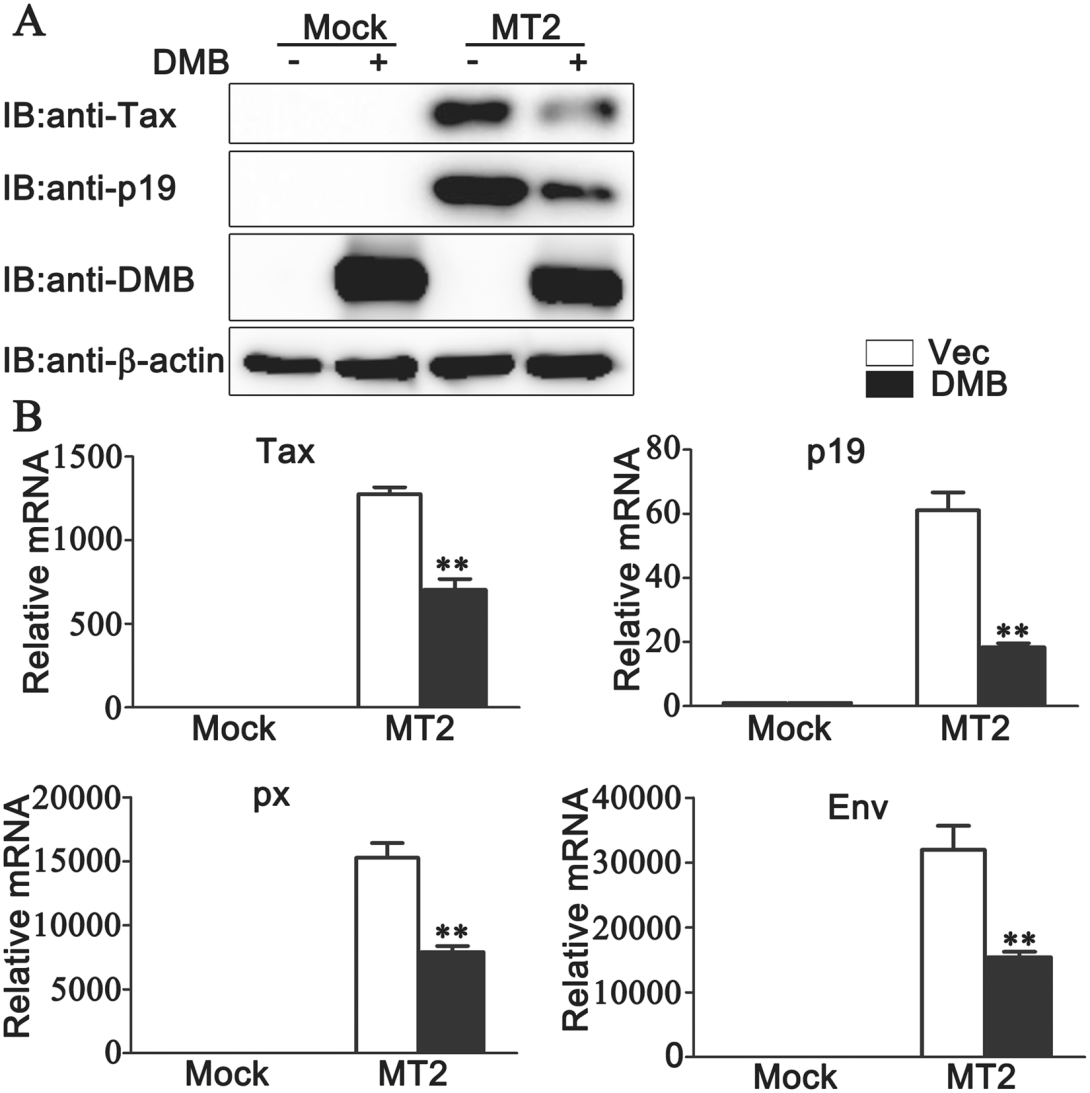
The effects of DMB overexpression on HTLV-1 protein expression. (**A**) Hela cells were transfected with empty vector (−) or pcDNA3.1-DMΒ (+). At 24 h after transfection, the cells were co-cultured with MT2 cells for another 24 h. Then the cells were washed with PBS three times to remove MT2 cells and lysed for immunoblot assays. (**B**) Hela cells were transfected with empty vector (−) or pcDNA3.1-DMΒ (+). At 24 h after transfection, the cells were co-cultured with MT2 cells for another 24 h. Then the cells were washed with PBS three times to remove MT2 cells and the real-time PCR analyses were performed. β-actin was used as a loading control in the immunoblot assays. The data were representative of three independent experiments and were presented as means ± SD (n = 3). **p  < 0.01.

Then we tried to determine the functions of DMΒ during HTLV-1 infection. Hela cells overexpressing DMB were co-cultured with MT2 cells and the expression levels of HTLV-1 proteins were investigated. Immunoblot assays demonstrated that the exogenous expression of DMB was associated with lower protein levels of HTLV-1 proteins Tax and p19 (Fig. 2A). Consistently, real-time PCR results indicated that the expression levels of HTLV-1 proviral transcripts for Tax, p19, Env and px were decreased in the presence of DMΒ (Fig. 2B). Taken together, these data suggest that the exogenous expression of DMΒ decreases HTLV-1 protein expression.

### Knockdown of DMB increases HTLV-1 protein expression

**Figure 3:**
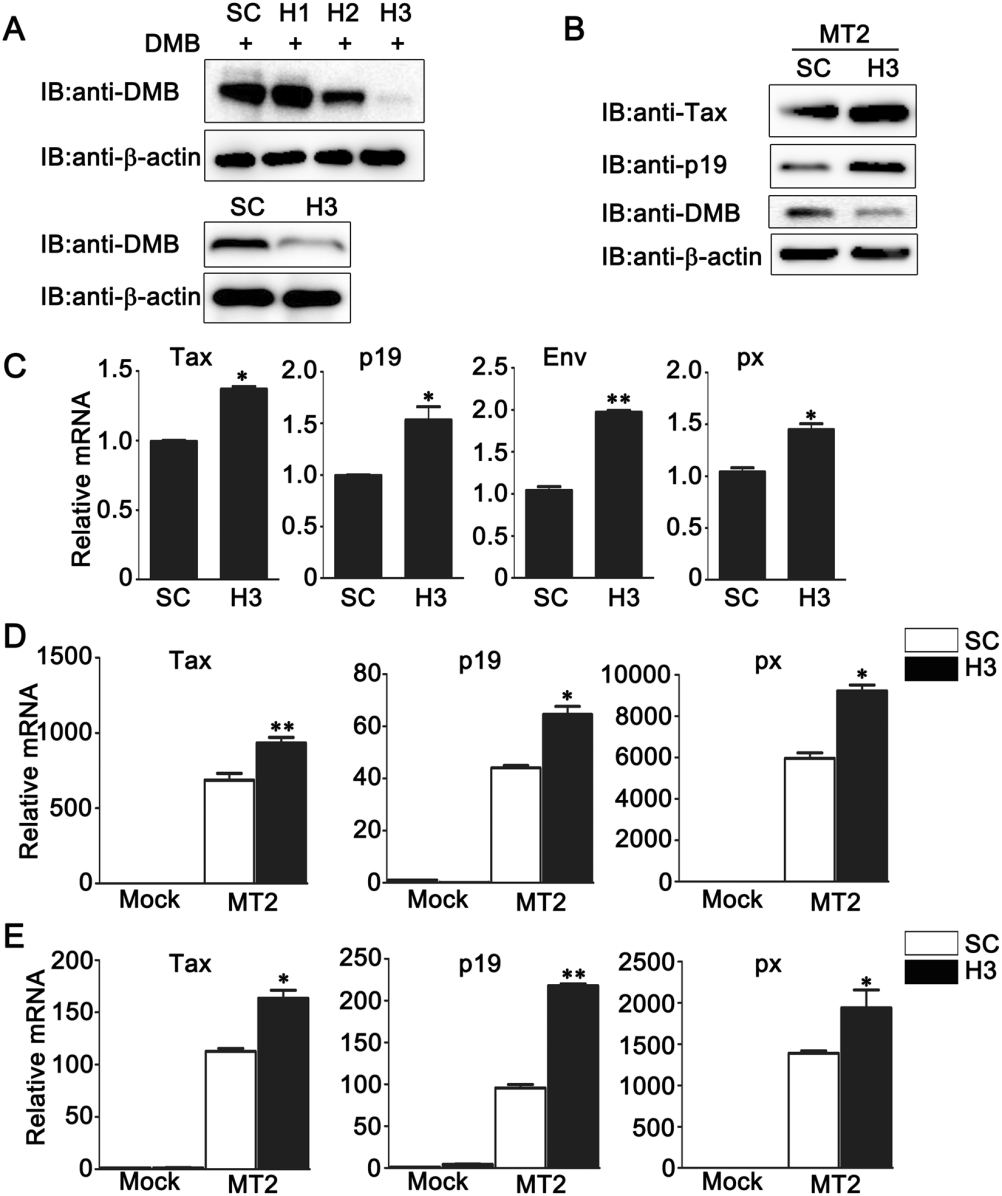
The effects of DMB knockdown on HTLV-1 protein expression. (**A**) HEK293T cells expressing pcDNA3.1-DMΒ (top) or MT2 cells (bottom) were transfected with control siRNA (SC) or DMB-specific siRNA (H1, H2, and H3). At 24 h after transfection, the cells were lysed for immunoblot assays. (**B**) MT2 cells were transfected with SC or H3. At 24 h after transfection, the cells were lysed for immunoblot assays. (**C**) MT2 cells were transfected with SC or H3. At 24 h after transfection, the cells were lysed for the real-time PCR analyses. (**D,E**) Hela (**D**) or PMA-THP1 (**E**) cells were transfected with SC or H3. At 24 h after transfection, the cells were co-cultured with MT2 cells for another 24 h. Then the cells were washed with PBS three times to remove MT2 cells and lysed for the real-time PCR analyses. β-actin was used as a loading control in the immunoblot assays. The data were representative of three independent experiments and were presented as means ± SD (n = 3). *p  < 0.05, **p  < 0.01.

Next, we examined whether endogenous DMΒ was involved in the regulation of HTLV-1 protein expression. We silenced endogenous DMB expression in MT2 cells by siRNA. We purchased three siRNA constructs and determined their effects on DMB expression. As shown in Fig. 3A, the #3 DMΒ-siRNA construct (H3) could markedly inhibit the expression of transfected pcDNA3.1-DMΒ in HEK293T cells and endogenous DMΒ in MT2 cells as suggested by immunoblot analysis. Then the H3 was used to silence the DMB expression and the effect of endogenous DMB expression on HTLV-1 protein expression was investigated. As shown in Fig. 3B, immunoblot assays demonstrated that the expressions of Tax and p19 were increased after the knockdown of DMB in MT2 cells. Consistently, after DMΒ was silenced in the MT2 cells, proviral transcription was monitored by real-time PCR assays and the results indicated that the expression levels of HTLV-1 proviral transcripts for Tax, p19, Env and px were higher in DMB-silenced MT2 cells (Fig. 3C). Similar results were observed in Jurkat and primary CD4+ T cells (see Supplementary Fig. S1). Next, we examined the role of DMΒ in HTLV-1 infected Hela or PMA-THP1 cells. After the knockdown of DMΒ, Hela or PMA-THP1 cells were co-cultured with MT2 cells and HTLV-1 protein expression was investigated. As shown in Fig. 3D and E, compared to cells transfected with control siRNA, real-time PCR assays indicated that the expression amounts of HTLV-1 proviral transcripts for Tax, p19 and px were increased after the knockdown of DMΒ in both HTLV-1 infected Hela (Fig. 3D) and PMA-THP1 (Fig. 3E) cells. Taken together, these results suggest that the knockdown of DMΒ increases HTLV-1 viral protein expression in all cell lines we have tested.

### DMB inhibits HTLV-1 infection induced autophagosome accumulation

**Figure 4:**
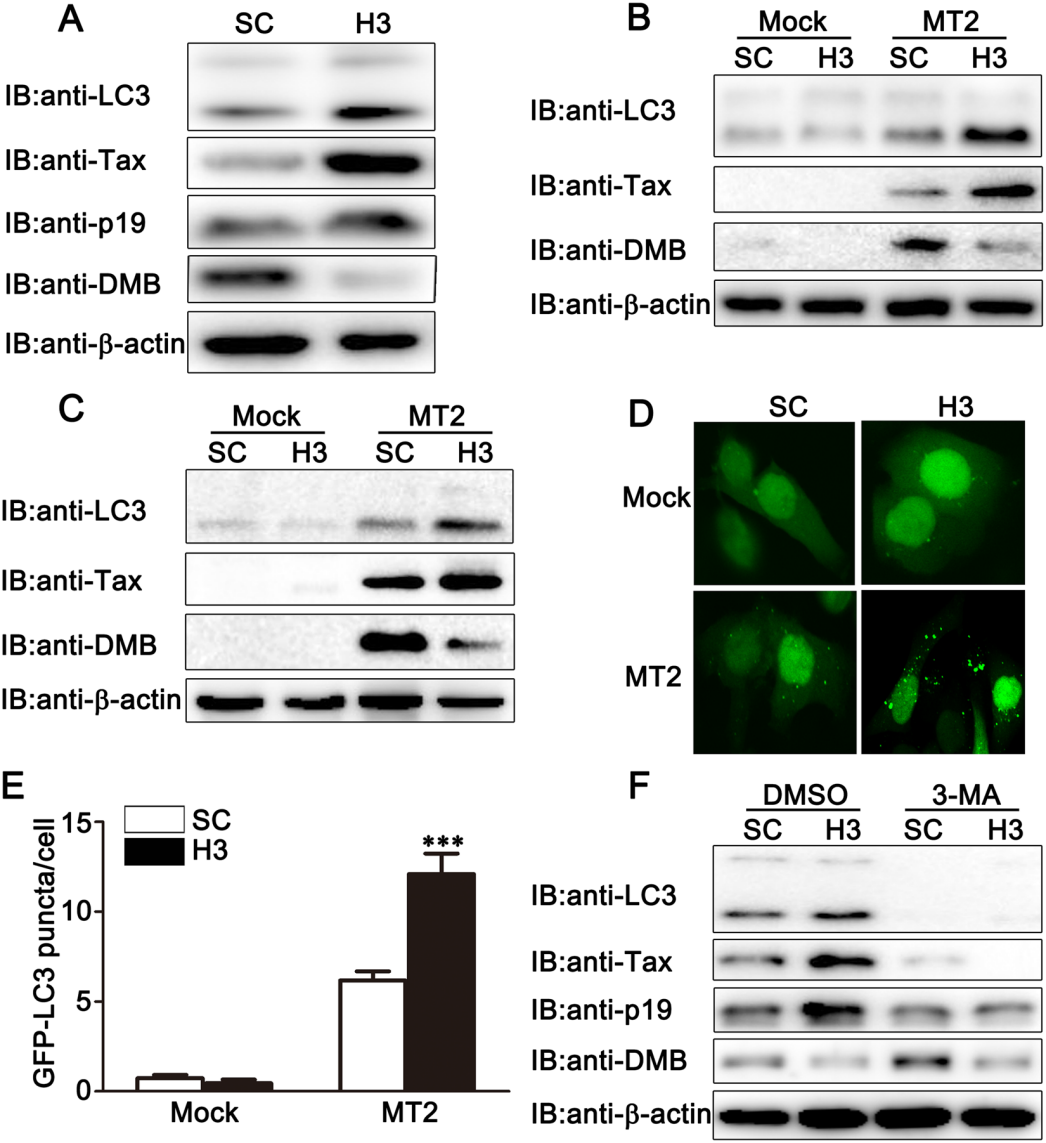
The effects of DMB on HTLV-1 induced autophagosome accumulation. (**A**) MT2 cells were transfected with SC or H3. At 24 h after transfection, the cells were lysed for immunoblot assays. (**B,C**) Hela (**B**) or PMA-THP1 (**C**) cells were transfected with SC or H3. At 24 h after transfection, the cells were co-cultured with MT2 cells for another 24 h. Then the cells were washed with PBS three times to remove MT2 cells and lysed for immunoblot assays. (**D**) Hela cells were transfected with EGFP-LC3. At 24 h after transfection, the cells were transfected with SC or H3. At 24 h after transfection, the cells were co-cultured with MT2 cells for another 24 h. Then the cells were washed with PBS three times to remove MT2 cells and confocal microscopy analyses were performed. (**E**) The number of LC3 puncta per cell from images as in (**D**) was counted from n > 400 cells for each treatment over 3 repeats. The bars represent mean ± SD. ***p <0.001. (**F**) Hela cells were transfected with SC or H3. At 24 h after transfection, the cells were pretreated with control DMSO or 5 mM 3-MA for 6 h. Then the cells were incubated with MT2 cells for 24 h in the presence of control DMSO or 5 mM 3-MA as indicated. The cells were washed with PBS three times to remove MT2 cells and lysed for immunoblot assays. β-actin was used as a loading control in the immunoblot assays. The data were representative of three independent experiments.

Given that type I IFNs play an important role in antiviral responses, we wondered whether DMB inhibited viral protein expression by regulating type I IFNs production. Hela cells were transfected with SC or H3 and then co-cultured with MT2 cells. At 24 h after co-culturation, the expression levels of IFNβ and IFN-responsive genes were examined by real-time PCR assays. The results suggested that the knockdown of DMB had no significant effects on HTLV-1 infection induced production of IFNβ and IFN-responsive genes (see Supplementary Fig. S2). It has been reported that HTLV-1 infection increases the accumulation of autophagosomes and that this accumulation increases HTLV-1 production^28^. Then we examined the effects of DMB on HTLV-1 induced autophagosome accumulation. DMB was silenced in MT2 cells and immunoblot assays were performed. We found that the knockdown of DMB increased the amount of LC3-II protein levels (Fig. 4A). Similar results were observed in Jurkat and primary CD4+ T cells (see Supplementary Fig. S1). Then we repeated the above analysis in MT2-co-cultured Hela and PMA-THP1 cells. After co-culture with MT2 cells, Hela (Fig. 4B) or PMA-THP1 (Fig. 4C) cells transfected with H3 displayed enhanced LC3-II expression and reduced p62 expression compared to the cells transfected with control siRNA. Consistently, the accumulation of LC3-II was increased in DMB-knockdown Hela cells compared to control cells after co-culture with MT2 cells as determined by GFP-LC3 assays (Fig. 4D and E). Finally, we determined the role of DMB in MT2-co-cultured cells in the presence of 3-Methyladenine (3-MA), which is believed to block the early stage of autophagy^15^. We found that DMB knockdown could not affect 3-MA inhibited LC3-II accumulation (Fig. 4F). Meanwhile, in 3-MA treated Hela cells, DMB knockdown could not increase HTLV-1 protein expression (Fig. 4F), suggesting that the effect of DMB on HTLV-1 protein expression was associated with autophagy. Taken together, these results suggest DMB inhibits HTLV-1 induced autophagosome accumulation and this effect is important to its role in regulating HTLV-1 protein expression.

### DMB affects HTLV-1 induced autophagosome accumulation and HTLV-1 protein expression in primary human monocytes

**Figure 5:**
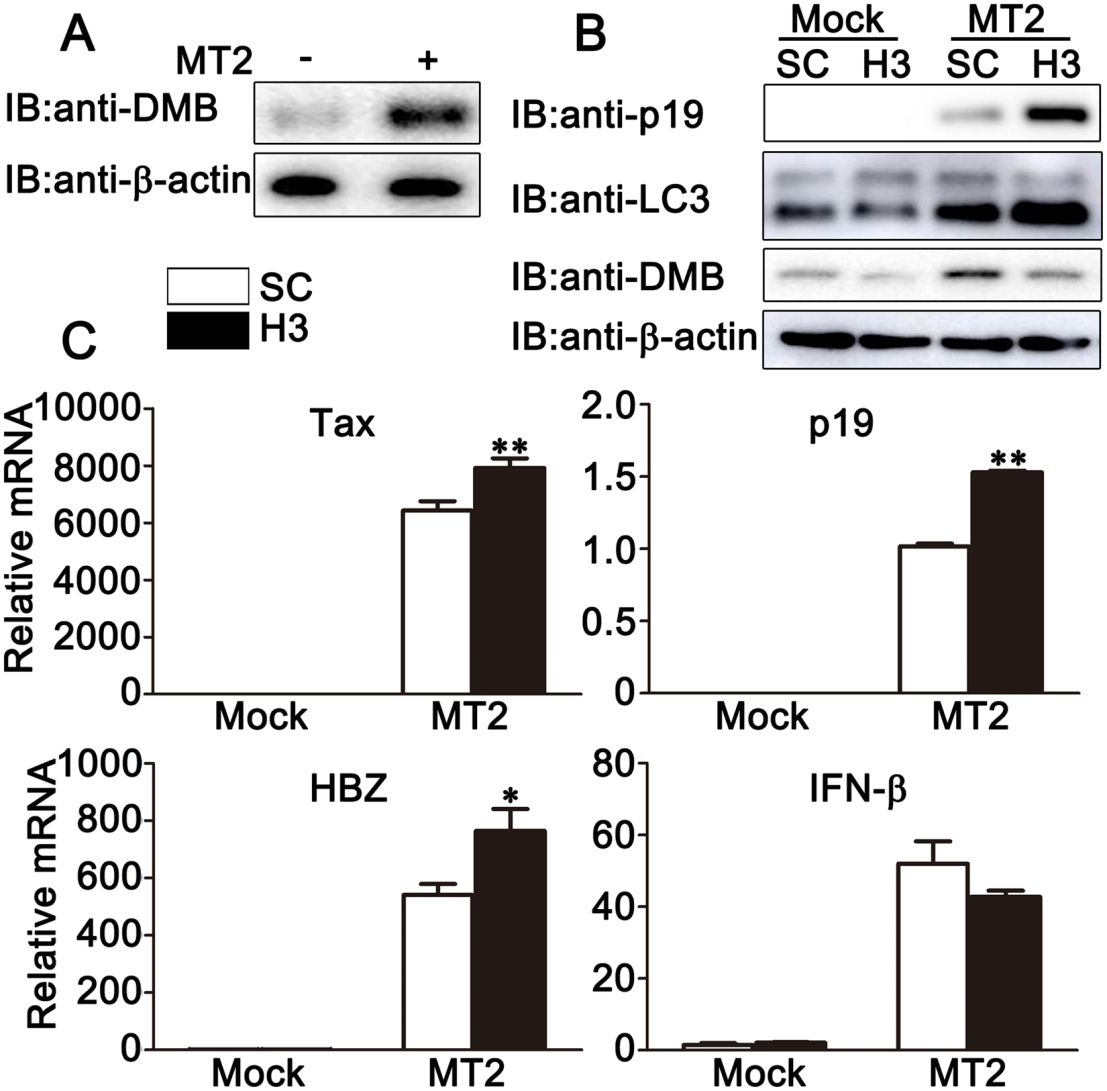
The effects of DMB knockdown on HTLV-1 induced autophagosome accumulation and HTLV-1 protein expression in primary human monocytes. (**A**) Human monocytes were co-cultured with MT2 cells for 24 h. Then the cells were washed with PBS three times to remove MT2 cells and lysed for immunoblot assays. (**B,C**) Human monocytes were transfected with SC or H3. At 24 h after transfection, the cells were co-cultured with MT2 cells for another 24 h. Then the cells were washed with PBS three times to remove MT2 cells and lysed for immunoblot analyses (**B**) or real-time PCR assay (**C**). β-actin was used as a loading control in the immunoblot assays. The data were representative of three independent experiments and were presented as means ± SD (n = 3). *p <0.05, **p <0.01.

To confirm the role of DMB during HTLV-1 infection in primary cells, we examined the effects of DMB in primary human monocytes after co-culture with MT2 cells. As shown in Fig. 5A, the expression of DMB was much higher in primary human monocytes after co-culture with MT2 cells. Then we silenced DMB expression in primary human monocytes by siRNA and investigated its role in HTLV-1 induced autophagosome accumulation and HTLV-1 protein expression. Western blot assays showed that DMB-silenced human monocytes had a higher expression level of p19 and an increased LC3-II level after co-culture with MT2 cells (Fig. 5B). Consistently, real-time PCR assays indicated that the expression of HTLV-1 proviral transcripts for Tax, p19 and HBZ was enhanced after the knockdown of DMB in MT2-co-cultured human monocytes (Fig. 5C). However, no significant difference was observed in the expression levels of IFN-β in DMB-silenced or control human monocytes after co-culture with MT2 cells (Fig. 5C). Taken together, these data suggest DMB affects HTLV-1 induced autophagosome accumulation and HTLV-1 protein expression in primary human monocytes.

### DMB interacts with ATG7

**Figure 6:**
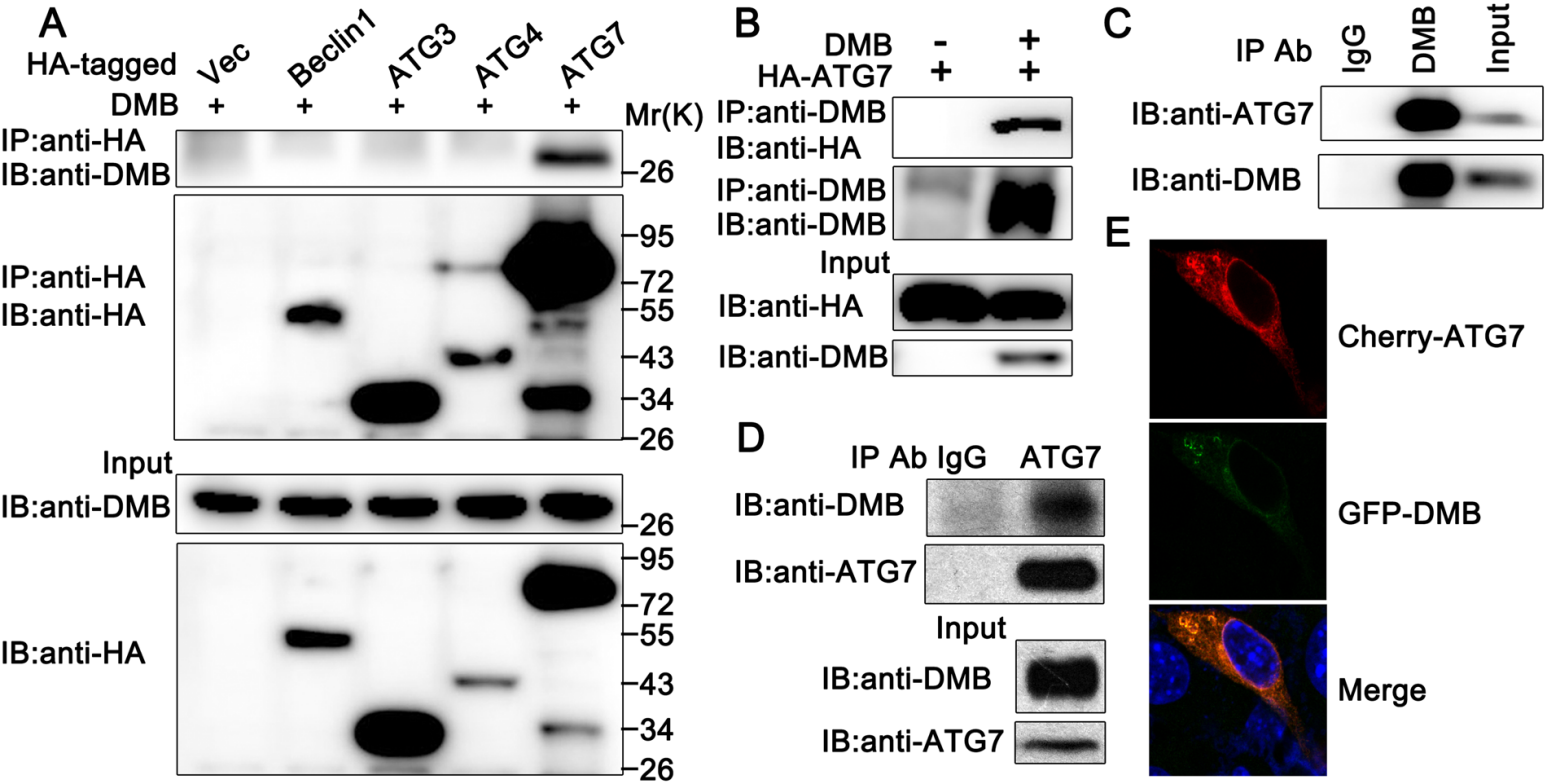
DMB interacts with ATG7. (**A**) HEK293T cells were transfected with indicated plasmids. The cell lysates were immunoprecipitated (IP) with anti-HA and immunoblotted (IB) with anti-HA or anti-DMB as indicated. (**B**) HEK293T cells were transfected with HA-tagged ATG7 and empty vector or pcDNA3.1-DMB. The cell lysates were immunoprecipitated (IP) with anti-DMB and immunoblotted (IB) with anti-DMB or anti-HA as indicated. (**C**) MT2 cells were immunoprecipitated with anti-ATG7 and immunoblotted with anti-DMB or anti-ATG7 as indicated. (**D**) PMA-THP1 cells were co-cultured with MT2 cells for 24 h. The cell lysates were then immunoprecipitated with anti-ATG7 and immunoblotted with anti-DMB or anti-ATG7 as indicated. (**E**) Confocal microscopy assays of Hela cells transfected with Cherry-ATG7 (Red) and EGFP-DMB (Green). The data were representative of three independent experiments.

To characterize the mechanism by which DMB regulated HTLV-1 induced autophagosome accumulation, we tested whether DMB was able to interact with the important ATG proteins involved in canonical autophagy. pcDNA3.1-DMΒ was transfected in HEK293T cells together with HA-tagged ATG proteins (including Beclin1, ATG3, ATG4 and ATG7). Subsequent coimmunoprecipitation experiments were performed and the data suggested only ATG7 bound to DMB (Fig. 6A and B). To confirm the endogenous interaction between ATG7 and DMB, MT2 cells were lysed and then subjected to immunoprecipitation analysis. The results suggested that endogenous DMB interacted with ATG7 (Fig. 6C). Similar results were observed in MT2-co-cultured PMA-THP1 cells (Fig. 6D). Consistently, confocal microscopy assays indicated that GFP-DMB was colocalized with Cherry-ATG7 in Hela cells (Fig. 6E). Taken together, these results suggest DMB interacts with ATG7.

Considered that DMB usually forms a heterodimeric protein with DMA, we also examined the effects of DMA on HTLV-1 infection. Hela cells overexpressing DMA were co-cultured with MT2 cells and the expression levels of Tax and p19 were investigated. Both immunoblot and real-time PCR results suggested that DMA had no significant effect on the expression levels of Tax and p19 (see Supplementary Fig. S3A and B). Moreover, coimmunoprecipitation data suggested only DMB bound to ATG7 and no significant interaction was detected between ATG7 and DMA (see Supplementary Fig. S3C). These results suggested that DMA might not be involved in DMB mediated regulation of HTLV-1 protein expression. We also examined the location of DMA and DMB. As shown in Supplementary Fig. S3D, DMB co-localized with DMA in lysosomes (suggested by the lysosome marker Lamp1). Interestingly, after ssDNA90 stimulation, part of the DMB protein translocated from lysosomes to the cytoplasmic and this part of DMB did not co-localize with DMA (see Supplementary Fig. S3D). Taken together, these data suggested that in addition to the role in the classical antigen-presenting process in the dimer with DMA, DMB might play a role in autophagy without the help of DMA.

### The association with ATG7 is important for the regulatory role of DMB during HTLV-1 infection

**Figure 7:**
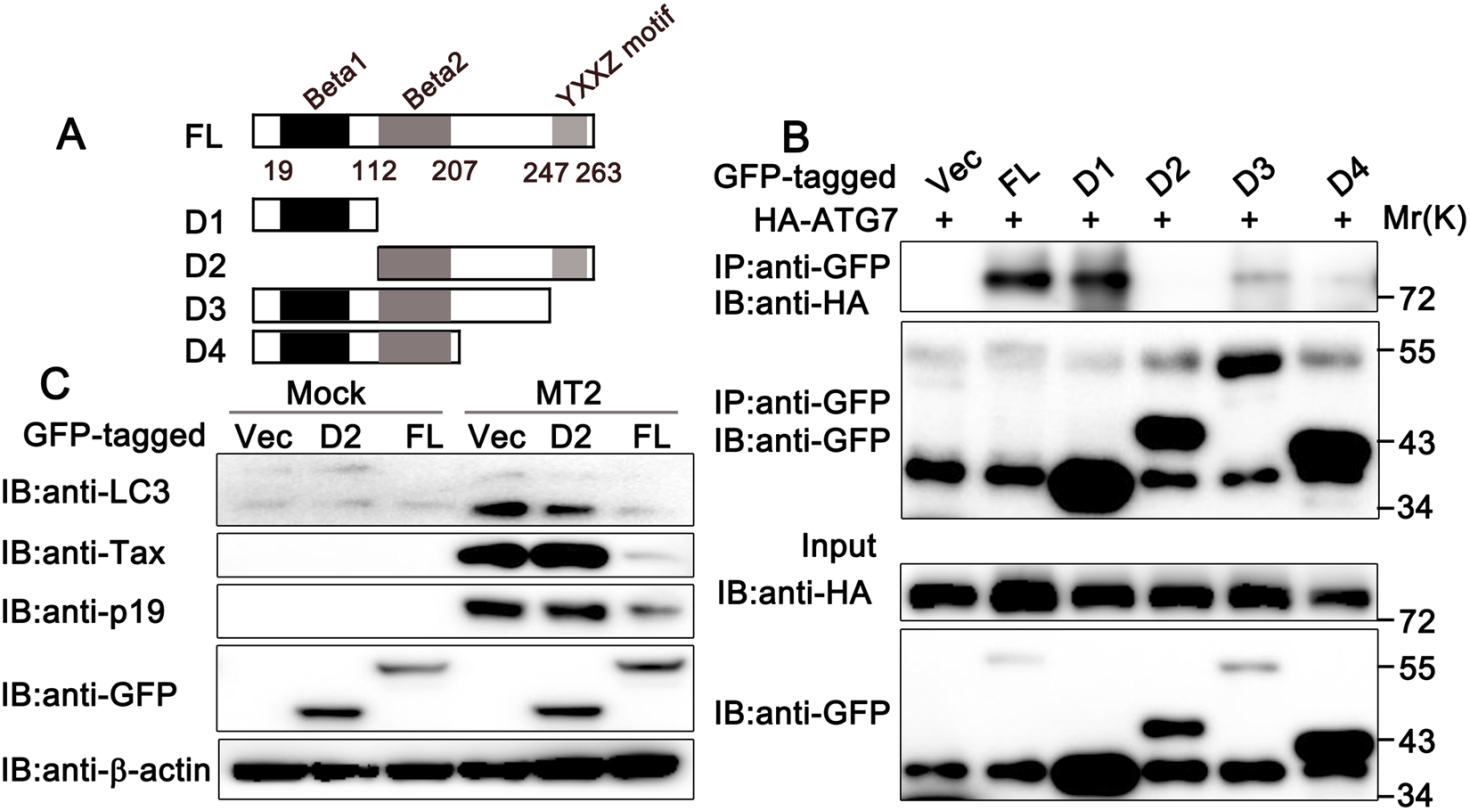
Domain mapping of the interaction between DMB and ATG7. (**A**) Schematic representation of DMB and its mutants. The full-length DMB contains Beta1 domain (19-112aa), Beta2 domain (113-207aa) and the YXXZ motif (247-263aa). FL, full length. (**B**) HEK293T cells were transfected with HA-ATG7 and indicated GFP-tagged plasmids. The cell lysates were immunoprecipitated with anti-GFP and immunoblotted with anti-HA or anti-GFP as indicated. (**C**) Hela cells were transfected with indicated plasmids. At 24 h after transfection, the cells were co-cultured with MT2 cells for another 24 h. Then the cells were washed with PBS three times to remove MT2 cells and lysed for immunoblot assays. β-actin was used as a loading control. The data were representative of three independent experiments.

To determine which part of DMB was essential for its interaction with ATG7, a series of GFP-tagged DMB deletion mutants, as described in Fig. 7A, were transfected into HEK293T cells with HA-tagged ATG7, and coimmunoprecipitation experiments were performed. As shown in Fig. 7B, the DMB truncation mutants D1 (aa1–112), D3 (aa1–247), and D4 (aa1–207) coimmunoprecipitated with ATG7, whereas D2 (aa113–263) did not, suggesting that the 1-112aa of DMB might be essential for its association with ATG7. Interestingly, although the truncation mutants D3 and D4 contained the 1-112aa of DMB, their ability of interacting with ATG7 was decreased compared to the D1 mutant and the full-length DMB. There was a possibility that the 112-207aa of DMB might block the binding site to some extent and the YXXZ motif (mediating the targeting to the lysosomal compartments) might enhance the interaction by affecting the location and 3D structure of DMB. Then, we determined whether the interaction between DMB and ATG7 was important for the regulatory function of DMB during HTLV-1 infection. Hela cells were transfected with full-length (FL) DMB or its D2 mutant and then co-cultured with MT2 cells. Immunoblot assays demonstrated that the D2 mutant had only a slight effect on LC3-II accumulation and HTLV-1 protein expression (Fig. 7C). Taken together, these results suggest that the 1-112aa of DMB is important for its interaction with ATG7 and this interaction is required for the regulatory function of DMB during HTLV-1 infection.

### DMB increases the acetylation of ATG7

**Figure 8:**
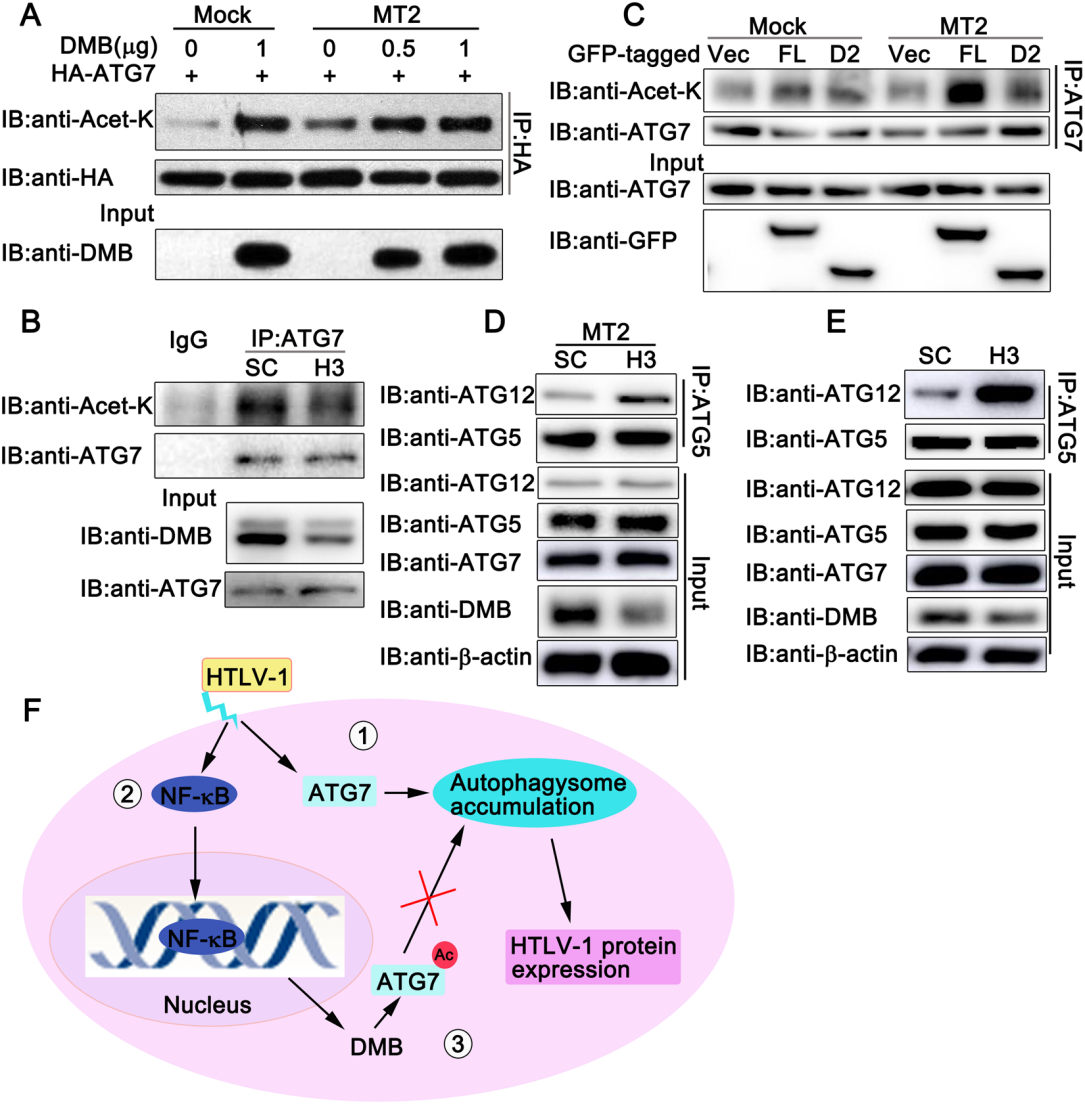
The effects of DMB on the acetylation of ATG7. (**A**) Hela cells were transfected with indicated plasmids for 24 h, and then co-cultured with or without MT2 cells for another 12 h. The cells were washed with PBS three times to remove MT2 cells and lysed. The cell lysates were immunoprecipitated with anti-HA and immunoblotted with anti-Acetylated-Lysine antibody or anti-HA as indicated. (**B**) MT2 cells were transfected with SC or H3. At 24 h after transfection, the cell lysates were immunoprecipitated with anti-ATG7 or immunoblotted with anti-Acetylated-Lysine antibody or anti-ATG7 as indicated. (**C**) Hela cells were transfected with indicated GFP-tagged plasmids for 24 h, and then co-cultured with or without MT2 cells for another 12 h. The cells were washed with PBS three times to remove MT2 cells and lysed. The cell lysates were immunoprecipitated with anti-ATG7 and immunoblotted with anti-Acetylated-Lysine antibody or anti-ATG7 as indicated. (**D**) PMA-THP1 cells were transfected with SC or H3 for 24 h, and then co-cultured with MT2 cells for another 24 h. The cells were washed with PBS three times to remove MT2 cells and lysed. The cell lysates were immunoprecipitated with anti-ATG5 and immunoblotted with the indicated antibodies. (**E**) MT2 cells were transfected with SC or H3. At 24 h after transfection, the cells were lysed. The cell lysates were immunoprecipitated with anti-ATG5 and immunoblotted with the indicated antibodies. β-actin was used as a loading control in the immunoblot assays. The data were representative of three independent experiments. (**F**) The schematic diagram of the interactions among HTLV-1, autophagy and DM. ①HTLV-1 infection induced the accumulation of autophagosome and this accumulation enhanced HTLV-1 protein expression. ②Meanwhile, HTLV-1 infection induced the expression of DMB. ③DMB expression increased the acetylation of ATG7, leading to decreased autophagy accumulation and lower HTLV-1 protein expression levels. Ac, acetylation.

Given the fact that the p300 promoted acetylation of ATG7 inhibits autophagy^35^, we tried to explore whether DMB could modulate the acetylation of ATG7. pcDNA3.1-DMΒ was cotransfected with HA-tagged ATG7, and the acetylation of ATG7 was examined in Hela cells co-cultured with MT2 cells or not. As shown in Fig. 8A, the exogenous expression of DMΒ promoted the acetylation of ATG7 in both MT2-co-cultured Hela cells and untreated Hela cells. We confirmed this result in endogenous conditions and found the knockdown of DMB decreased the acetylation of ATG7 in MT2 cells (Fig. 8B). Moreover, the D2 mutant of DMB, which was unable to interact with ATG7, had a slight effect on the acetylation of ATG7 in MT2-co-cultured Hela cells (Fig. 8C). Because ATG7 could activate ATG12 and promote the formation of ATG12-ATG5 complex^13^, we examined whether DMB impaired the ATG12-ATG5 conjugation. Coimmunoprecipitation experiments indicated that the knockdown of DMB enhanced the endogenous ATG12-conjugation no matter in MT2-co-cultured PMA-THP1 cells (Fig. 8D) or MT2 cells (Fig. 8E). Then we tried to explore the mechanism by which DMB enhance the acetylation. It has been reported that Sirtuin 1 (Sirt1) can interact with ATG7 and directly deacetylate ATG7^36^. So we tried to determine whether DMB modulated the acetylation of ATG7 via Sirt1. We inhibited the Sirt1 activity by EX527 in Hela cells and found that DMB had little effect on the acetylation of ATG7 in the presence of EX527 (see Supplementary Fig. S4A), suggesting the effect of DMB on the acetylation of ATG7 may be dependent on Sirt1 activity. We assumed that DMB might have the potential to regulate the acetylation of ATG7 by disrupting its interaction with Sirt1. To address this issue, we examined the effect of exogenous expressed DMB on the association between ATG7 and Sirt1 by competitive coimmunoprecipitation. The results suggested that DMB was able to decrease the association of Sirt1 with ATG7 (see Supplementary Fig. S4B). Then we explored the effect of endogenous DMB on the interaction between Sirt1 and ATG7. The knockdown of DMB in MT2 or MT2-co-cultuled PMA-THP1 cells increased the amount of Sirt1 that coimmunoprecipitated with ATG7 (see Supplementary Fig. S4C and D), suggesting DMB might regulate the acetylation of ATG7 by inhibiting the interaction between Sirt1 and ATG7. Taken together, these results suggest DMB inhibits autophagosome accumulation by increasing the acetylation of ATG7.

## Discussion

It is well known that DM is a kind of non-peptide binding MHC-class-II molecules and acts as an enzyme to catalyze peptide exchange required for efficient loading of endosomal peptides onto MHC-II molecules^37^. However, the role of DM in the overall immune response remains unclear, although several papers have reported DM may function in the development of several autoimmune diseases, such as type I diabetes^38,39^. Our findings suggested that DMB inhibited HTLV-1 induced autophagosome accumulation and decreased the expression levels of HTLV-1 proteins.

Our results showed that HTLV-1-positive T-cell lines had a strong expression of DMB and HTLV-1 infection induced DMΒ expression in Hela, PMA-THP1 or primary human monocytes. Further studies indicated that the overexpression of DMB inhibited HTLV-1 protein expression and DMB knockdown was associated with higher levels of HTLV-1 protein expression. Because of the importance of type I IFN in the host anti-viral responses, the effect of DMB on type I IFN production was examined. However, no significant change was observed with or without the presence of DMB. These results drove us to find other possible mechanism to explain the role of DMB in HTLV-1 infection.

As increasing evidence has shown that autophagy plays a complex role in viral infection, it is worth noting that HTLV-1 infection accumulates autophagosomes to benefit the virus replication^28^. Thus, it was reasonable for us to suspect the inhibitory role of DMB on HTLV-1 protein expression might have some relation with autophagy. We used LC3-II as the marker of autophagy and examined the effect of DMB on HTLV-1 induced autophagy. Our findings confirmed the hypothesis by three steps. Firstly, DMB inhibited the autophagosome accumulation in MT2 cells. Secondly, DMB inhibited the autophagosome accumulation induced by HTLV-1 infection in Hela, PMA-THP1 or primary human monocytes. Thirdly, when we used 3-MA to block the early stage of autophagy, we found that DMB knockdown lost the abilities to promote autophagosome accumulation and to increase HTLV-1 viral protein expression, suggesting that the regulatory role of DMB in autophagosome accumulation was important to its effect on HTLV-1 protein expression.

How could a non-classical MHC-II protein regulate autophagosome accumulation? As an E1-like enzyme, ATG7 has been reported to be required in both ubiquitin-like LC3 and ATG12 conjugation systems and is essential to autophagosome formation. Thus, it is a good target for regulation. Our findings indicated that DMB interacted with ATG7 through co-immunoprecipitation and co-localization experiments. Using a serious of DMB deletion mutants, we found DMB aa1-112 was essential for the association of DMB with ATG7. Furthermore, the DMB D2 mutant (without aa1-112) almost lost the ability to regulate autophagosome accumulation and HTLV-1 protein expression, suggesting that the interaction between DMB and ATG7 was essential to the regulatory role of DMB in autophagy and HTLV-1 infection.

Acetylation is one of the important posttranslational modifications for the regulation of autophagy, which could affect ATG gene expression or activity^40^. It has been reported that deacetylation of ATG5, ATG7 and ATG8 by the NAD-dependent deacetylase Sirt1 could stimulate autophagy^36^. Here, we showed that DMB affected the acetylation of ATG7, thereby playing an inhibitory role in autophagosome accumulation. The overexpression of DMB promoted the acetylation of ATG7 in both MT2-co-cultured Hela cells and untreated Hela cells, whereas the knockdown of DMB decreased the acetylation of ATG7 in MT2 cells. Although our data have suggested DMB might regulate the acetylation of ATG7 by inhibiting the interaction between Sirt1 and ATG7, further studies about the underlying molecular mechanism are needed.

It is worth noting that the detailed mechanisms about how autophagosome accumulation can increase the abundance of HTLV-1 protein remain to be clarified. It has been revealed that HTLV-1 protein Tax, which plays an important role in regulating HTLV-1 replication, blocks the fusion of autophagosomes to lysosomes and prevented its degradation of HTLV-1 protein in the autophagy-lysosome pathway^28^. One may think that the inhibition of autophagosome-lysosome fusion by Tax results in increased autophagosome accumulation and decreased degradation of HTLV-1 protein, leading to increased HTLV-1 proteins abundance. Interestingly, our data showed that the DMB expression inhibited autophagosome accumulation at the early phase of the autophagy pathway and resulted in impaired HTLV-1 protein expression. These results indicated that the autophagosome formation might provide convenience for HTLV-1 protein expression. It is possible that HTLV-1 infection promotes the autophagosome formation to benefit its replication. Meanwhile the Tax protein inhibits the degradation of autophagosome to make the best use of this benefit. Further studies are needed to clarify the detailed mechanisms.

Together, our study demonstrated that DMB modulated autophagosome accumulation by regulating the acetylation of ATG7, thereby affecting the expression of HTLV-1 protein (Fig. 8F). Our research may expand our understanding of the regulators in host anti-viral responses and suggest a new role of non-canonical MHC-II protein in autophagy.

## Methods

### cDNA constructs and reagents

Human HLA-DMB was amplified by PCR using cDNA from MT2 cells, and subsequently cloned into pcDNA3.1 vector. The deletion mutants of DMB were amplified by PCR and subcloned into pEGFP vector. Human ATG3, ATG4, ATG7, and Beclin1 were amplified by PCR using cDNA from Hela cells, and cloned into a pcDNA3.1-HA vector. ATG7 was subcloned into a pmCherry vector. pENTER-Flag-DMA plasmid was obtained from Vigene Biosciences. Myc-Sirt1 plasmid was a generous gift of Xiaofei Zhu (Xinxiang Medical University, Henan, China). The 90-base-long HTLV-1 ssDNA90 is the reverse complement of the 5′UTR region (315–404) of complete HTLV-1 genome (NCBI) and was synthesized from the Sangon Biotech. The sequence was as follows: CTGTGTACTAAATTTCTCTCCTGGAGAGTGCTATAGAATGGGCTGTCGCTGGCTCCGAGCCAGCAGAGTTGCCGGTACTTGGCCGTGGGC.

The following antibodies were used for immunoblot or immunoprecipitation analysis: anti-HA (CO-MMS-101R, Covance), anti-Flag (F3165, Sigma-Aldrich), anti-DMB (ab131273, Abcam), anti-HTLV-1 p19 (ab9080, Abcam), anti-LAMP1 (ab24170, Abcam), anti-Tax (sc-57872, Santa Cruz Biotechnology), anti-IFI16 (sc-8023, Santa Cruz Biotechnology), anti-LC3A/B (4108, Cell Signaling Technology), anti-ATG5 (12994, Cell Signaling Technology), anti-ATG7 (8558, Cell Signaling Technology), anti-ATG12 (4180, Cell Signaling Technology), anti-Acetylated-Lysine (9441, Cell Signaling Technology), anti-Sirt1 (2493, Cell Signaling Technology), anti-GFP (66002-1-Ig, Proteintech), anti-Myc (66004-1-Ig, Proteintech), anti-cGAS (26416-1-AP), Proteintech) and anti-β-actin (60008-1, Proteintech).

The 3-MA (M9281) was obtained from Sigma-Aldrich. All the chemical inhibitors were obtained from Selleck. The PMA (S1819) was purchased from Beyotime Biotechnology.

### Cell culture and transfection

HEK293T and Hela cells were cultured in DMEM. MT2, C8166, Jurkat and THP1 cells were grown in RPMI 1640. PBMCs were enriched from donor blood using Ficoll density gradient separation and human monocytes were separated by their adherence to the culture plate in RPMI 1640. Primary CD4^+^ T cells were isolated from PBMCs by FACS screening, and cultured in RPMI 1640 in the presence of IL-2. All cells were supplemented with 10% FBS (Gibco), 4mM L-glutamine, 100U/ml penicillin, and 100U/ml streptomycin under humidified conditions with 5% CO_2_ at 37 °C. Transfection of HEK293T and Hela cells was performed with Lipofectamine 2000 (Invitrogen) according to the manufacturer’s instructions.

### Immunoprecipitation and immunoblot analysis

Immunoprecipitation and immunoblot analysis were performed as described previously^41^. In short, HEK293T, Hela, THP1 or MT2 cells were transfected with various combinations of plasmids or siRNA. At 24 h after the transfection, the cell lysates were prepared in lysis buffer containing 1.0% (vol/vol) Nonidet P40, 20 mM Tris-HCl, pH 8.0, 10%(vol/vol) glycerol, 150 mM NaCl, 0.2 mM Na3VO4, 1 mM NaF, 0.1 mM sodium pyrophosphate and a protease inhibitor ‘cocktail’ (Roche). After centrifugation for 20 min at 14,000 g, supernatants were collected and incubated with the indicated antibody together with protein A/G Plus-agarose immunoprecipitation reagent (sc-2003, Santa Cruz Biotechnology) at 4 °C for 3 h or overnight. After three washes, the immunoprecipitants were boiled in SDS sample buffer for 10 min and analyzed by immunoblot. For endogenous coimmunoprecipitation experiments, the cell lysates of MT2 cells or THP1 cells were incubated with indicated antibodies and analyzed by immunoblot.

### Real-time PCR

Total RNA was extracted from the cultured cells with TRIzol reagent (Invitrogen) as described by the manufacturer. All gene transcripts were quantified by real-time PCR with SYBR Green qPCR Master Mix using a 7500 Fast real-time PCR system (Applied Biosystems). The relative fold induction was calculated using the 2^−△△Ct^ method. Gene-specific primer sequences were as described^42^ or as follow:

p19,

Forward, 5′-CACCCCTTTCCCTTTCATTCACGA-3′,

Reverse, 5′- CCGGCCGGGGTATCCTTTT-3′,

Env,

Forward, 5′-CCATCGTTAGCGCTTCCAGCCCC-3′,

Reverse, 5′- CGGGATCCTAGCGTGGGAACAGGT-3′;

px(This region is between env and the 3′-long terminal repeat. The PCR product of this pair of primers is the common part of HTLV-1 proviral transcripts for Tax, Rex and p27),

Forward, 5′- CAAAGTTAACCATGCTTATTATCAGC-3′,

Reverse, 5′-ACACGTAGACTGGGTATCCGAA-3′

HBZ,

Forward, 5′- AACTGTCTAGTATAGCCATCA -3′;

Reverse, 5′- CAAGGAGGAGGAGGAAGCTGTGC -3′

ISG15,

Forward, 5′- ATGGGCTGGGACCTGACCGG -3′;

Reverse, 5′- TTAGCTCCGCCCGCCAGGCT -3′

IP-10,

Forward, 5′- TTCCTGCAAGCCAATTTTGTC -3′;

Reverse, 5′- TCTTCTCACCCTTCTTTTTCATTGT -3′

RANTES,

Forward, 5′- TACACCAGTGGCAAGTGCTC -3′;

Reverse, 5′- ACACACTTGGCGGTTCTTTC -3′

### RNA interference

HLA-DMB-Stealth-RNA interference was designed by the Invitrogen BLOCKiT RNAi Designer. The small interfering RNA (siRNA) sequences used were as follows:

H1,

Forward, 5′-UCCUUCAACAAGGAUCUGCUGACCU-3′,

Reverse, 5′-AGGUCAGCAGAUCCUUGUUGAAGGA-3′;

H2,

Forward, 5′-GGACAUACCAGACCCUCUCCCAUUU-3′,

Reverse, 5′-AAAUGGGAGAGGGUCUGGUAUGUCC-3′;

H3,

Forward, 5′-ACUCCUCUUCCUGGGUCCAAUUAUU-3′,

Reverse, 5′-AAUAAUUGGACCCAGGAAGAGGAGU-3′.

The negative control siRNA was purchased from Invitrogen (Catalog no.12935300).

STING-Stealth-RNA interference was designed by the Invitrogen BLOCKiT RNAi Designer. The small interfering RNA (siRNA) sequences used were as follows:

Forward, 5′-GGCCCGGAUUCGAACUUACAAUCAG-3′;

Reverse, 5′-CUGAUUGUAAGUUCGAAUCCGGGCC-3′.

Ku70-Silencer Select Pre-designed siRNA was obtained from Invitrogen. The small interfering RNA (siRNA) sequences used were as follows:

Forward, 5′-GACAUAUCCUUGUUCUACATT-3′;

Reverse, 5′-UGUAGAACAAGGAUAUGUCAA-3′;

cGAS-Stealth-RNA interference was designed by the Invitrogen BLOCKiT RNAi Designer. The small interfering RNA (siRNA) sequences used were as follows:

Forward, 5′-GCACGUGAAGAUUUCUGCACCUAAU-3′;

Reverse, 5′-AUUAGGUGCAGAAAUCUUCACGUGC-3′.

IFI16-Stealth-RNA interference was designed by the Invitrogen BLOCKiT RNAi Designer. The small interfering RNA (siRNA) sequences used were as follows:

Forward, 5′-UCAAUCAGCUUUGCUCACAAACUAA-3′;

Reverse, 5′-UUAGUUUGUGAGCAAAGCUGAUUGA-3′

THP1, Hela, MT2, Jurkat, primary CD^+^ T cells or primary human monocytes were transfected with siRNA using Lipofectamine 2000 according to the manufacturer’s instructions. At 24 h after transfection, the cells were used for further experiments.

### Confocal microscopy

Hela cells were transfected with the indicated plasmids. At 24 h after transfection, cells were fixed with 4% PFA in PBS and permeabilized with Triton X-100 and then blocked with 1% BSA in PBS. Nuclei were stained with DAPI.

### Statistics

The data were presented as the means ± SD from at least three independent experiments. The statistical comparisons between the different treatments were performed using the unpaired Student t test, and *p* < 0.05 was considered statistically significant.

## Electronic supplementary material

Supplemental materials
