# Supplementary material for: HLA-DMB restricts human T-cell leukemia virus type-1 (HTLV-1) protein expression via regulation of ATG7 acetylation
Source: Sci Rep. 2017 Oct 31;7:14416. doi: 10.1038/s41598-017-14882-z (PMC5663917; doi:10.1038/s41598-017-14882-z)
Supplement: Supplementary file 1 — Supplemental materials [file 41598_2017_14882_MOESM1_ESM.pdf]

HLA-DMB restricts human T-cell leukemia virus type-1 (HTLV-1) protein expression via regulation of ATG7 acetylation

Jie Wang[1,6#], Di Song[1,6#], Yanzi Liu[2], Guangjian Lu[3], Shuai Yang[1], Lu Liu[1], Zhitao Gao[4], Lingling Ma[1], Zhixiang Guo[1], Chenguang Zhang[1], Hui Wang[5]* and Bo Yang[1,6]*

[1]Henan Collaborative Innovation Center of Molecular Diagnosis and Laboratory Medicine, School of Laboratory Medicine, Xinxiang Medical University, Xinxiang 453003, Henan Province, China

[2]Department of Laboratory Medicine, the Third Affiliated Hospital of Xinxiang Medical University, Xinxiang 453003, Henan Province, China

[3]Clinical Laboratory, the First Affiliated Hospital of Xinxiang Medical University, Weihui 453100, Henan Province, China

[4]Department of Immunology/Department of Bio-therapeutic, Institute of Basic Medicine, School of Life Sciences, PLA Medical School, Chinese PLA General Hospital, 28 Fuxing Road, Beijing, 100853, China

[5]Research center for immunology, School of laboratory medicine, Xinxiang Medical University

[6]Xinxiang assegai medical laboratory institute, Xinxiang 453003, Henan Province, China

Supplementary Fig. S1

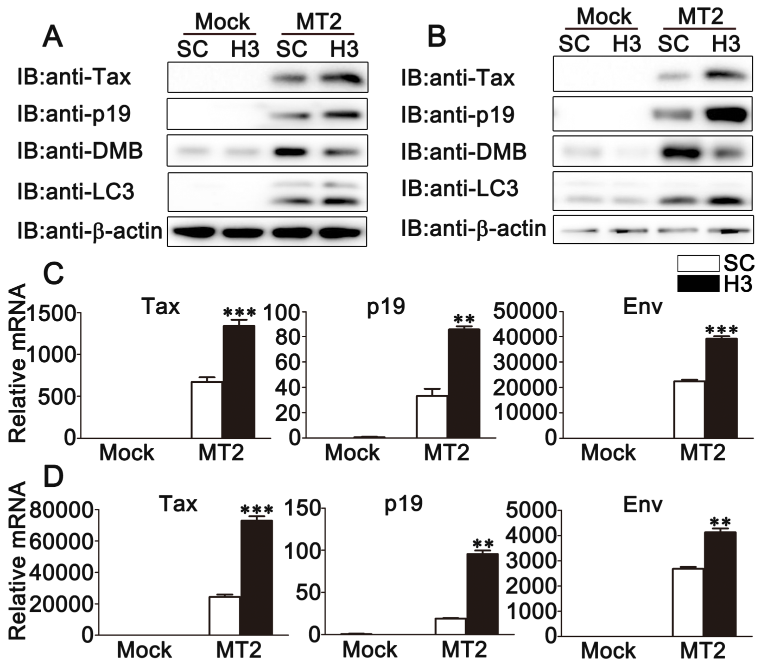

Supplementary Fig. S1 The effects of DMB knockdown on HTLV-1 protein expression in Jurkat and primary CD4$^+$ T cells (A, B) Jurkat (A) or primary CD4$^+$ T cells (B) were transfected with SC or H3. At 24h after transfection, the cells were labeled with CFSE and co-cultured with MT2 cells for another 24h. Then the CFSE positive cells were collected by FACS screening and lysed for immunoblot assays. β-actin was used as a loading control. (C, D) Jurkat (C) or primary CD4$^+$ T cells (D) were transfected with SC or H3. At 24h after transfection, the cells were labeled with CFSE and co-cultured with MT2 cells for another 24h. Then the CFSE positive cells were collected by FACS screening and lysed for the real-time PCR analyses. The data were representative of three independent experiments and were presented as means ± SD (n = 3). $^{**}p < 0.01$, $^{***}p < 0.001$.

Supplementary Fig. S2

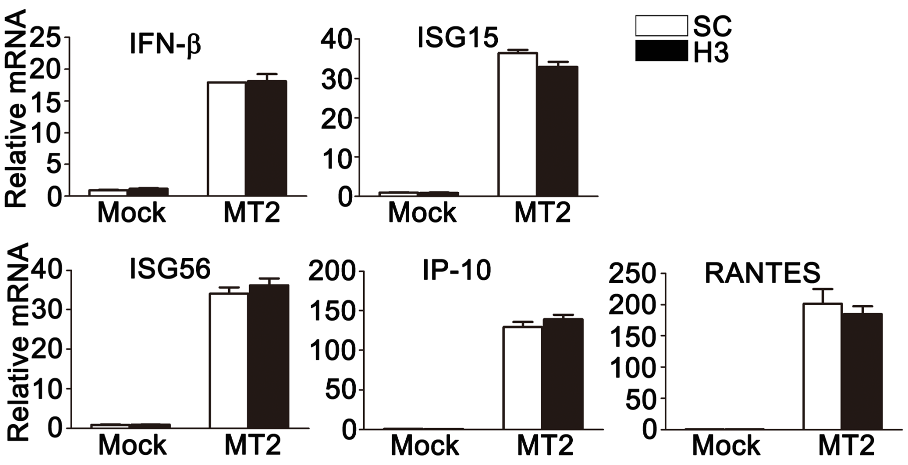

Supplementary Fig. S2 The effects of DMB knockdown on HTLV-1 induced IFN-β and IFN-responsive genes production in Hela cells. Hela cells were transfected with SC or H3. At 24h after transfection, the cells were co-cultured with MT2 cells for 24h. Then the cells were washed with PBS three times to remove MT2 cells and lysed for the real-time PCR analyses. The data were representative of three independent experiments and are presented as means ± SD (n = 3).

Supplementary Fig. S3

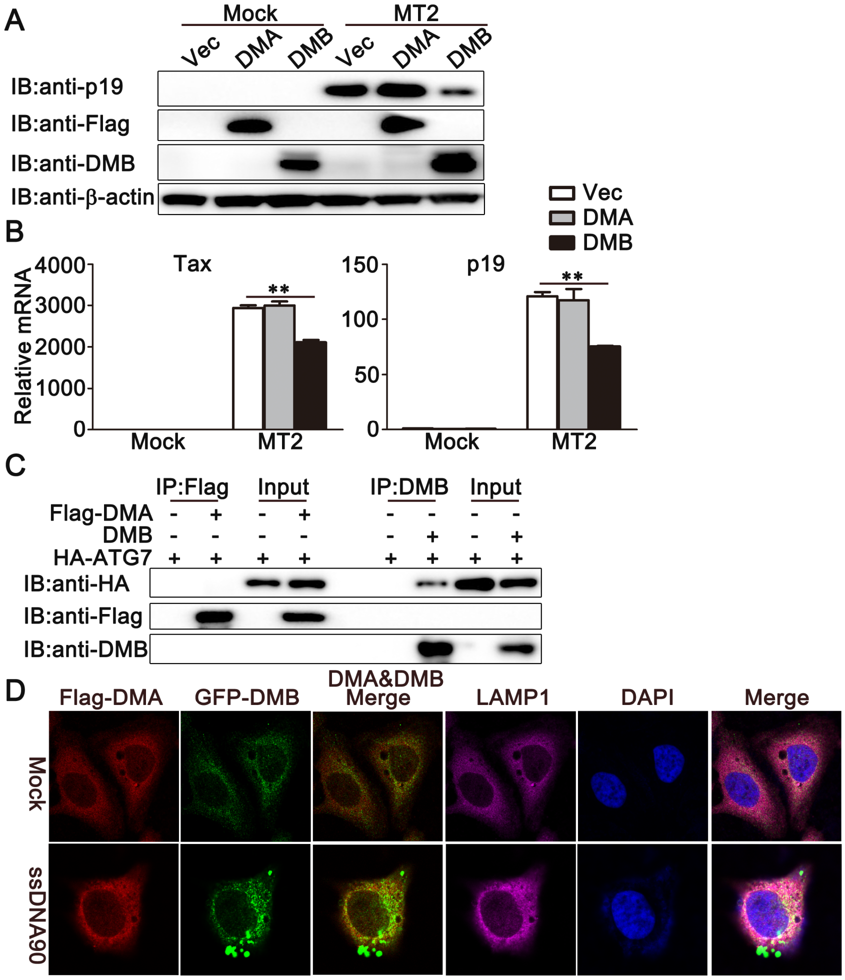

Supplementary Fig. S3 The effects of DMA overexpression on HTLV-1 protein expression (A, B) Hela cells were transfected with empty vector, pcDNA3.1-DMB or pENTER-Flag-DMA. At 24h after transfection, the cells were co-cultured with MT2 cells for another 24h. Then the cells were washed with PBS three times to remove MT2 cells and lysed for immunoblot analyses (A) or real-time PCR assay (B). β-actin was used as a loading control. (C) HEK293T cells were transfected with indicated plasmids. At 24h after transfection, the cell lysates were immunoprecipitated (IP) with anti-Flag or anti-DMB as indicated and immunoblotted (IB) with anti-Flag, anti-DMB or anti-HA as indicated. (D) Hela cells were transfected with expressing plasmids for Flag-DMA and GFP-DMB. At 24h after transfection, Hela cells were transfected with 1 ug ssDNA90 or left untreated for another 8h. Then the cells were prepared for confocal microscopy. Nuclei were stained with DAPI. The data were representative of three independent experiments and were presented as means±SD (n = 3). ***p*$<$0.01.

Supplementary Fig. S4

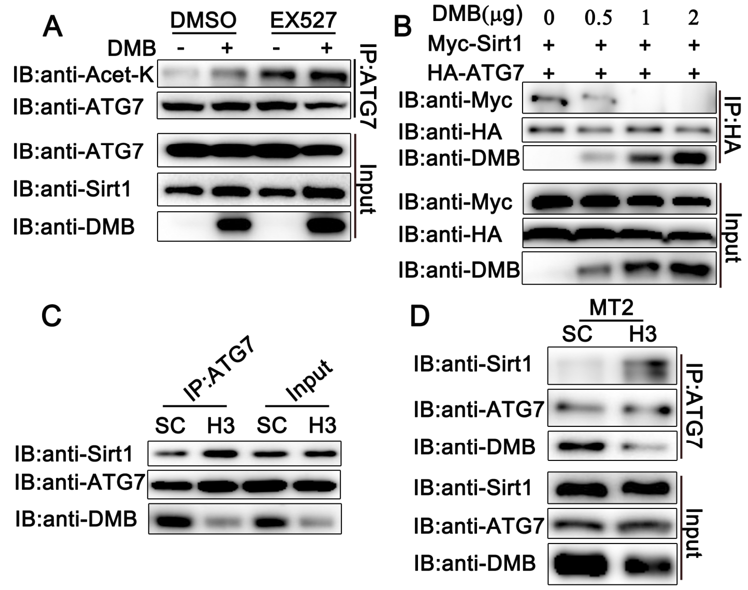

Supplementary Fig. S4 The effects of DMB on the Sirt1 mediated deacetylation of ATG7. (A) Hela cells were transfected with empty vector (-) or pcDNA3.1-DMB (+), and then treated with 20 uM EX527 for 24h. The cell lysates were immunoprecipitated with anti-ATG7 and immunoblotted with anti-Acetylated-Lysine, anti-Sirt1, anti-ATG7 or anti-DMB as indicated. (B) HEK293T cells were transfected with indicated plasmids for 24h. The cell lysates were immunoprecipitated with anti-HA and immunoblotted with anti-Myc, anti-HA or anti-DMB as indicated. (C) MT2 cells were transfected with SC or H3. At 24h after transfection, the cell lysates were immunoprecipitated with anti-ATG7 and immunoblotted with anti-Sirt1, anti-ATG7 or anti-DMB as indicated. (D) PMA-THP1 cells were transfected with SC or H3 for 24h, and then co-cultured with or without MT2 cells for another 24h. The cells were washed with PBS three times to remove MT2 cells and lysed. The cell lysates were immunoprecipitated with anti-ATG7 and immunoblotted with anti-Sirt1, anti-ATG7 or anti-DMB as indicated. The data were representative of three independent experiments.

Supplementary Fig. S5

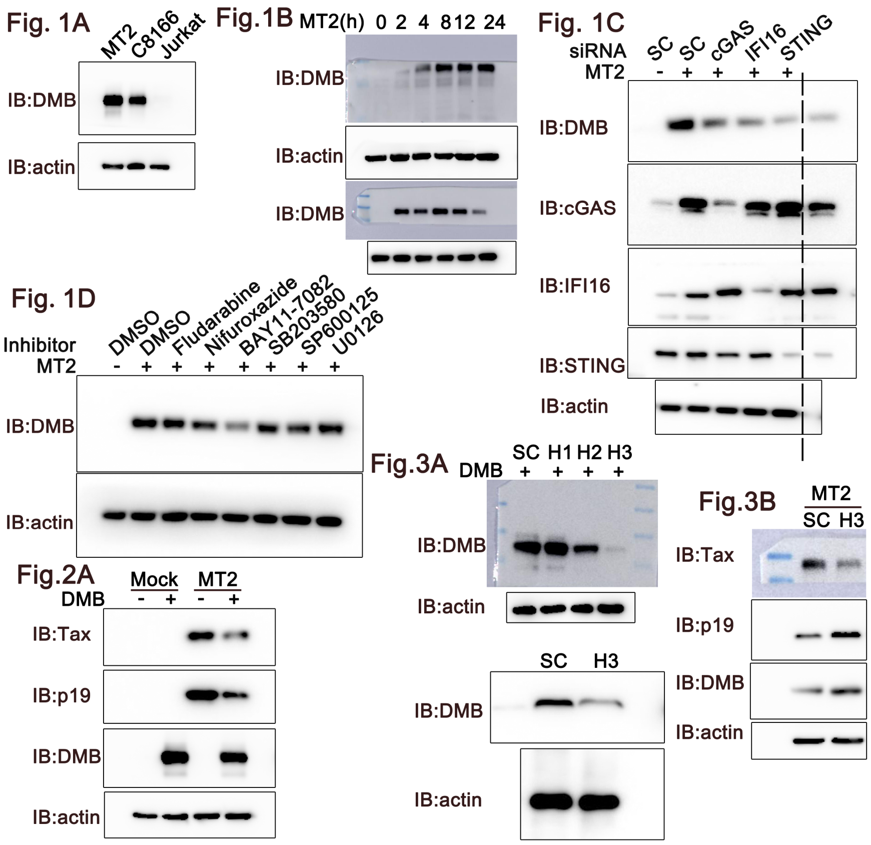

Supplementary Fig. S5 Full-length images of blots and gels presented in the main figures (Figure 1, Figure 2 and Figure 3)

Supplementary Fig. S6

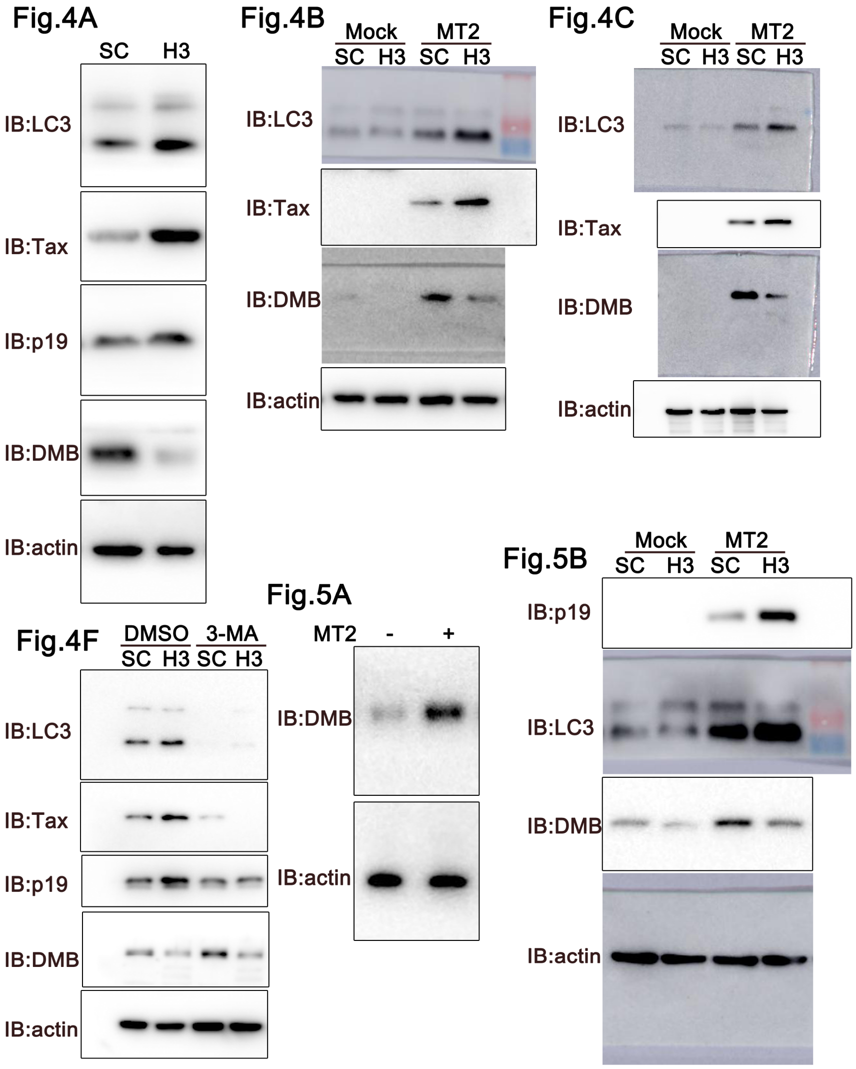

Supplementary Fig. S6 Full-length images of blots and gels presented in the main figures (Figure 4 and Figure 5)

Supplementary Fig. S7

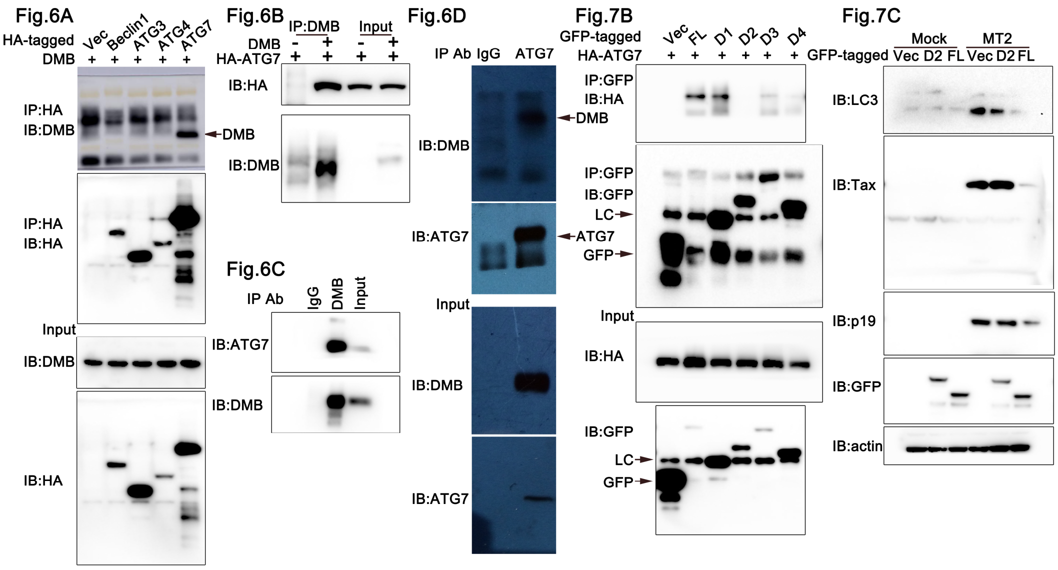

Supplementary Fig. S7 Full-length images of blots and gels presented in the main figures (Figure 6 and Figure 7)

Supplementary Fig. S8

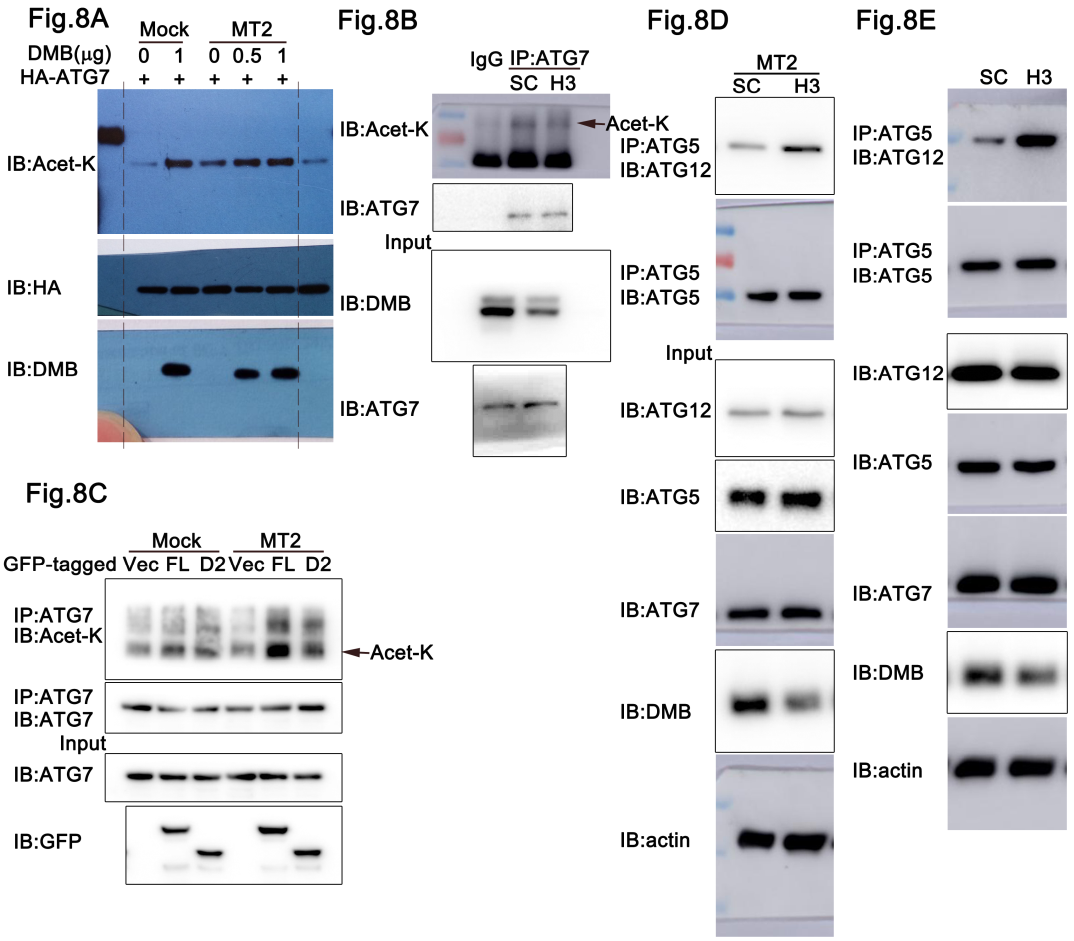

Supplementary Fig. S8 Full-length images of blots and gels presented in the main figures (Figure 8)
